# From self-construal to trait- and state-fear of missing out: the roles of need to belong and active social media use

**DOI:** 10.3389/fpsyg.2026.1743687

**Published:** 2026-07-23

**Authors:** Lihua Zuo, Jian Mao, Lili Yang, Haini Wang, Zihan Xie

**Affiliations:** 1 Mental Health and Counseling Center, Guangdong Ocean University, Zhanjiang, China; 2 Department of Psychology/Research Center of Adolescent Psychology and Behavior, School of Education, Guangzhou University, Guangzhou, China; 3 The Affiliated Zhanjiang School of Guangdong Experimental Middle School, Zhanjiang, China

**Keywords:** active social media use, fear of missing out (FoMO), interdependent self-construal, need to belong, state-FoMO, trait-FoMO

## Abstract

**Introduction:**

Fear of missing out (FoMO) has become a common psychological experience in the context of widespread social media use. Building on the trait–state FoMO framework, the present study examined how interdependent self-construal is associated with trait- and state-FoMO through need to belong, and whether active social media use moderates these associations.

**Methods:**

The hypotheses were tested among Chinese college students using a questionnaire survey in Study 1 and a scenario-based experimental design in Study 2.

**Results:**

The results showed that interdependent self-construal was not robustly or directly associated with trait-FoMO, but was indirectly associated with trait-FoMO through need to belong. In contrast, interdependent self-construal showed a more direct association with state-FoMO and was also indirectly associated with state-FoMO through need to belong. Active social media use attenuated the positive association between need to belong and state-FoMO, whereas this moderating effect was not observed for trait-FoMO.

**Discussion:**

This study provides preliminary evidence for the combined roles of individual psychological factors and social media behaviors in FoMO. It contributes to clarifying the differences between trait- and state-FoMO and offers important implications for reducing FoMO while preserving its psychosocial functions.

## 1 Introduction

In today’s highly interconnected world, a psychological phenomenon known as “Fear of Missing Out” (FoMO) has become increasingly prevalent. Its main cognitive and behavioral manifestations are a strong desire to stay informed about others’ activities and frequent checking of social media (SM) (Elhai et al., 2020b). While FoMO is not a formal psychopathological diagnosis but rather a common everyday experience, its strong links to problematic SM use, anxiety, depression, and other adverse mental health consequences have drawn significant scholarly interest (Brailovskaia and Margraf, 2024; Elhai et al., 2020b; Fioravanti et al., 2021). Existing research indicates that FoMO is primarily influenced by factors such as personality traits, psychological needs, and patterns of SM use (Gao et al., 2026; Tandon et al., 2021; Zhang et al., 2024). Recently, Dogan (2019) has linked self-construal with FoMO, demonstrating that how individuals perceive themselves has an important impact on FoMO.

Moreover, as research has advanced, scholars have gradually realized that treating FoMO as a single construct may be overly simplistic. Similar to distinctions drawn in psychology between traits such as anxiety and self-esteem, FoMO has been conceptualized as comprising two related yet distinct dimensions: Trait-FoMO and state-FoMO (Wegmann et al., 2017). Trait-FoMO is regarded as a personality disposition that reflects a persistent concern about missing out on others’ experiences, experienced across various times and situations. By contrast, state-FoMO refers to a transient cognitive bias characterized explicitly by the fear of missing out on information online. Researchers generally agree that examining the differences between trait and state-FoMO is a highly valuable research question in this field (Elhai et al., 2020b; Tandon et al., 2021). In light of this, the present study will separately examine the effects and mechanisms of interdependent self-construal on trait and state-FoMO, and further explore whether the underlying mechanisms of these two types of FoMO differ. This will not only advance understanding of the mechanisms through which self-construal shapes FoMO and yield practical implications for reducing FoMO to enhance psychosocial functioning, but also provide fresh empirical support for the trait–state perspective of FoMO.

### 1.1 Interdependent self-construal and FoMO

Self-construal is defined as the tendency of individuals to understand themselves in relation to a particular referential framework (Hofmann and Hinton, 2014). Markus and Kitayama (1991) distinguished between independent and interdependent self-construals, representing typical self-construals in Western individualistic cultures (independent) and Eastern collectivistic cultures (interdependent). Independent self-construal highlights autonomy and a preference for separation from others, whereas interdependent self-construal focuses on relational connectedness, with self-representations often framed within interpersonal interactions (Markus and Kitayama, 1991). Extensive research indicates that independent and interdependent self-construals are not opposite poles of one dimension but can coexist in the same individual, with one orientation usually being more salient (Brewer and Gardner, 1996). Furthermore, while self-construal tendencies emerge as stable personality traits shaped by cultural contexts, they can also be situationally primed, such that the dominant self-construal shifts depending on contextual cues (Hofmann and Hinton, 2014; Trafimow et al., 1991).

Interdependent self-construal has been suggested as an important individual-level correlate of FoMO (Dogan, 2019). A defining feature of FoMO is the strong urge to maintain connections with people in individuals’ social networks (Elhai et al., 2020b). Compared with independent self-construal, which emphasizes autonomy, individuals with interdependent self-construal view others as part of the self and are more eager to maintain connectedness; thus, they experience FoMO out of fear of disconnection (Markus and Kitayama, 1991; Dogan, 2019). Second, empirical studies have found a moderate positive correlation between interdependent self-construal and FoMO (Dogan, 2019; Li et al., 2023). Experimental research has also shown that participants primed with interdependent self-construal report significantly higher levels of FoMO than those in the control group (Dogan, 2019).

### 1.2 The mediating role of the need to belong

According to the need-to-belong theory, people have a universal motivation to pursue and sustain at least a minimal level of enduring, positive, and meaningful social bonds—referred to as the need to belong (Baumeister and Leary, 1995). As a basic psychological need, belongingness plays a vital role in shaping cognition, affect, and behavior (Baumeister and Leary, 1995). Individuals with a stronger need to belong are more eager to maintain connections with others and show greater curiosity and attentiveness toward others’ activities, which may lead them to experience higher levels of FoMO (Baumeister and Leary, 1995; Wang et al., 2018). Research has found that the strength of the need to belong significantly predicts the level of FoMO (Beyens et al., 2016; Wang et al., 2018), and experimental studies have further shown that individuals with higher FoMO levels are more sensitive to social acceptance cues (Lai et al., 2016).

Dogan (2019) suggested that the need to belong may serve as an important mediating variable in the relationship between interdependent self-construal and FoMO. While belongingness is considered a basic psychological need, there are individual differences in its intensity (Leary et al., 2013). As mentioned earlier, self-construal theory distinguishes two types: compared with independent self-construal, which emphasizes autonomy, interdependent self-construal places greater importance on relational connectedness, making the establishment and maintenance of positive ties more crucial for such individuals. Thus, the intensity of the need to belong will likely vary depending on one’s self-construal orientation. Cross-sectional studies have found that interdependent self-construal significantly predicts the need to belong (Chang, 2015). Moreover, compared with the independent self-construal priming group, individuals primed with interdependent self-construal report a stronger need to belong (Chen and Yang, 2017). In sum, interdependent self-construal may affect FoMO by altering the intensity of one’s need to belong.

### 1.3 The moderating role of active social media use

According to self-determination theory, the satisfaction of basic psychological needs plays an important role in individuals’ self-regulation and emotional experiences (Ryan and Deci, 2000). Przybylski et al. (2013) applied this framework to explain FoMO as a negative affective experience associated with deficits in basic psychological need satisfaction or with hindered self-regulation when such needs remain unmet. Empirical studies have also shown that higher satisfaction of basic psychological needs is associated with lower levels of FoMO (Przybylski et al., 2013; Xie et al., 2018). Therefore, it is important to distinguish between the intensity of belongingness needs and the satisfaction of these needs. A strong need to belong indicates a heightened motivation to seek social connection, but whether this motivation develops into FoMO may depend on whether individuals experience sufficient interpersonal responsiveness and social connection. Recent experimental research further supports this view by showing that FoMO is rooted not merely in missing enjoyable events, but in perceived missed social bonding and concerns about the possible negative implications of missing out for future social belonging. Moreover, reaffirming one’s social belonging by recalling prior social connection can temporarily reduce FoMO (Rifkin et al., 2025). These findings suggest that when social connection is psychologically affirmed or satisfied, the link between belongingness needs and FoMO may be weakened.

Nowadays, online social networking has become an integral part of most people’s lives. Reports indicate that as of February 2025, there are 5.24 billion SM users worldwide, accounting for 63.9% of the global population (Petrosyan, 2025). A major survey in China showed that 99.39% (*n* = 5,118) of college students access SM every day (Chen et al., 2022). The uses and gratifications theory posits that SM is important in fulfilling individuals’ psychological needs (Malik et al., 2016). SM use can be divided into active and passive (Verduyn et al., 2015). Active use refers to behaviors involving direct interaction with others on SM, including sending messages or sharing links with friends, posting updates, or commenting on others’ posts (Verduyn et al., 2017).

Active SM use may provide one behavioral context in which belongingness needs are partially or temporarily satisfied. Unlike passive browsing, active SM use involves interpersonal exchange and may help individuals maintain social ties, obtain interpersonal feedback, perceive online network responsiveness, and experience a sense of being socially connected. Previous studies have shown that active SM use can maintain and enhance individuals’ offline and online social relationships, increase connectedness and interpersonal satisfaction (Mao et al., 2023), and facilitate the acquisition of social support and social capital (Iannone et al., 2018; Valkenburg and Peter, 2009; Trepte et al., 2018; Lin et al., 2022). Experimental evidence also suggests that increasing Facebook status updates can reduce loneliness by making individuals feel more connected to their friends (Deters and Mehl, 2013). More recent survey evidence further indicates that active SM use is positively associated with perceived online network responsiveness, which in turn predicts higher perceived social support, lower loneliness, and higher life satisfaction (Yue et al., 2024). These studies collectively indicate that active SM use may help satisfy or affirm individuals’ belongingness needs by providing social feedback and a temporary sense of connection. It can thus be inferred that, at the same level of belongingness, individuals with higher levels of active SM use may experience less FoMO than those with lower active use, as their need to belong is more fully satisfied. Put differently, active SM engagement may serve as a moderator in the link between the need to belong and FoMO.

### 1.4 Trait- and state- FoMO

Fear of missing out is multidimensional, with Wegmann et al. (2017) introducing the trait–state dual-structure of FoMO for the first time. A dispositional vulnerability at the personality level fundamentally characterizes Trait-FoMO. It refers to a persistent, stable inclination across situations, indicating an individual’s generalized anxiety about missing out on others’ experiences. Those with high trait-FoMO, similar to individuals with elevated trait anxiety, exhibit a more sensitive alert system to potential cues of missing social information (Przybylski et al., 2013; Wegmann et al., 2017). State-FoMO represents a situational cognitive–emotional reaction, specifically reflecting the experience of FoMO during SM use in contemporary studies (Wegmann et al., 2017; Röttinger et al., 2021). Studies show a moderate correlation between trait and state-FoMO; trait-FoMO tends to develop into state-FoMO, and conversely, state-FoMO can exacerbate trait-FoMO (Röttinger et al., 2021; Mao et al., 2023). However, trait-FoMO appears to reflect deficits in psychological needs and jealous tendencies, and is more closely associated than state-FoMO with anxiety, depression, loneliness, and interpersonal sensitivity (Balta et al., 2020; Mao et al., 2023; Röttinger et al., 2021; Yoosefi et al., 2025). Mao et al. (2023) found that interpersonal satisfaction was significantly associated with trait-FoMO but not with state-FoMO, and that trait-FoMO, rather than state-FoMO, significantly positively predicted loneliness among college students. Two other studies indicate that mental health issues such as depression and anxiety are associated with trait-FoMO but not significantly with state-FoMO (Balta et al., 2020; Wegmann et al., 2017). In contrast, state-FoMO is more closely related to SM usage behaviors. Frequent SM use directly increases state-FoMO rather than trait-FoMO, and elevated state-FoMO is a direct contributor to problematic SM use (Li et al., 2020; Li et al., 2024; Röttinger et al., 2021). Additionally, a study reported that active SM use reduces trait-FoMO while increasing state-FoMO (Mao and Zhang, 2023). Overall, limited evidence suggests that trait and state-FoMO are closely linked yet exhibit distinct differences.

### 1.5 The present study

Based on self-construal theory, the need-to-belong theory, and related empirical research, this study develops a moderated mediation model to explore how interdependent self-construal influences FoMO. Specifically, need to belong is conceptualized as reflecting the motivational intensity of social connection needs, whereas active SM use is considered a behavioral context that may partially satisfy or buffer such needs through interpersonal interaction, social feedback, and a temporary sense of social connection. Drawing on the trait–state FoMO framework, the study further examines whether interdependent self-construal has differential associations and mechanisms of influence on trait versus state-FoMO.

**Figure 1:**
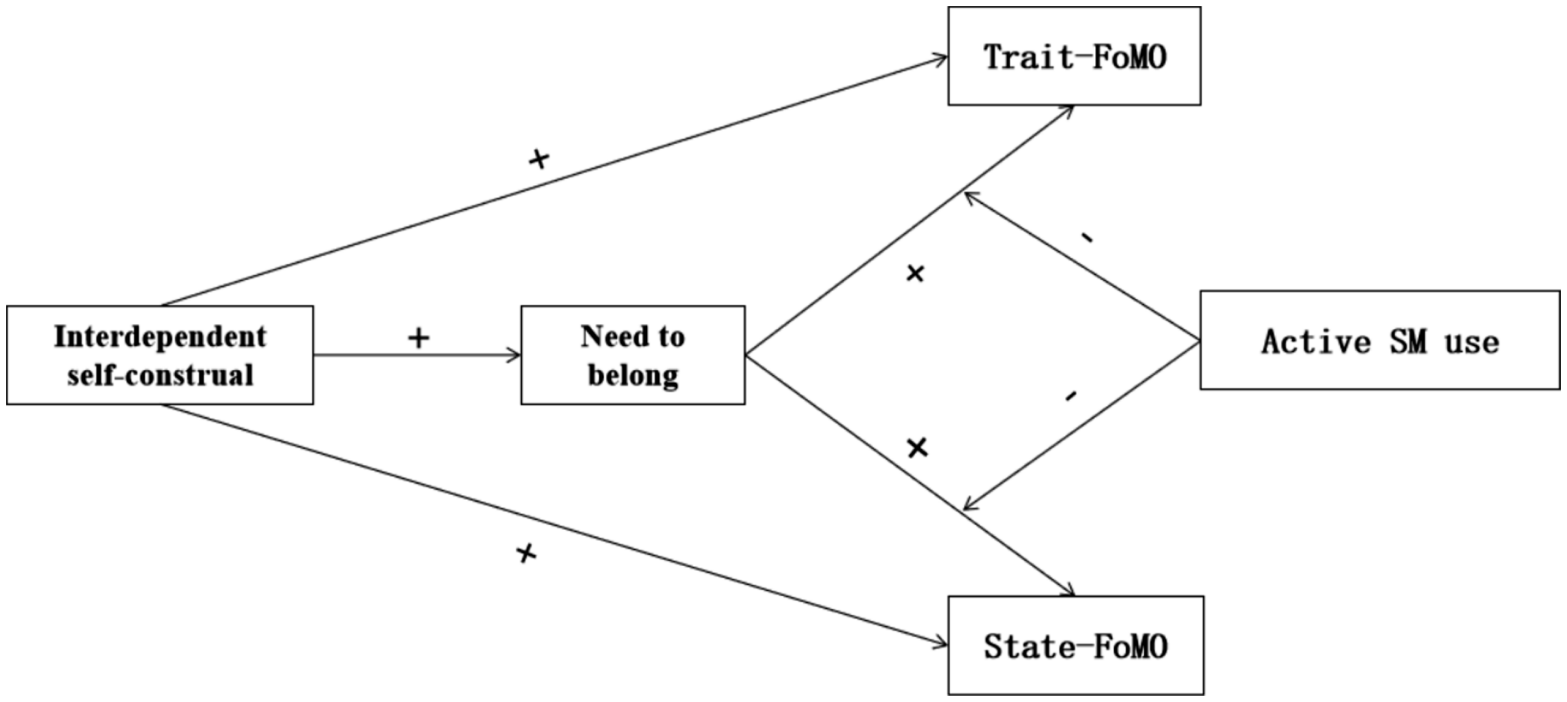
Hypothesis model.

As reviewed above, trait-FoMO reflects a relatively stable and generalized tendency to worry about missing rewarding experiences, whereas state-FoMO is more situation-sensitive and closely tied to online social contexts. Therefore, although interdependent self-construal and need to belong may be associated with both forms of FoMO, their relative roles in trait-FoMO and state-FoMO remain an open empirical question. By contrast, because active SM use mainly provides immediate interpersonal feedback, perceived responsiveness, and a temporary sense of social connection in online contexts, its buffering role may be more evident for state-FoMO than for trait-FoMO. Accordingly, the following hypotheses (Figure 1 shows the hypothetical model.) and research question were proposed:

*H1*: Interdependent self-construal is positively associated with trait-FoMO and state-FoMO.

*H2*: Need to belong mediates the associations between interdependent self-construal and trait-FoMO/state-FoMO.

*H3*: Active SM use moderates the second half of the pathway from interdependent self-construal to FoMO via the need to belong, such that the positive association between the need to belong and FoMO is weaker among individuals with higher levels of active SM use than among those with lower levels of active SM use.

*H3a*: The moderating role of active SM use is expected to be more evident for state-FoMO than for trait-FoMO.

RQ1: Do the direct and indirect associations involving interdependent self-construal and the need to belong differ between trait-FoMO and state-FoMO?

Finally, to enhance the robustness of the findings, two studies were conducted. Study 1 used a survey method to examine the associations among the main variables and to preliminarily test the proposed moderated mediation model. Study 2 employed a scenario-based experimental design to manipulate self-construal and active SM use, providing complementary evidence for the model under more controlled conditions.

## 2 Study 1: questionnaire survey

### 2.1 Methods

#### 2.1.1 Participants and procedure

Using convenience sampling, 387 undergraduate students (from first to fourth year) with at least 1 year of SM experience (e.g., QQ, WeChat Moments) were recruited from three universities in two central Chinese cities (Nanchang, Ganzhou city) for a questionnaire survey. The survey was administered online via the Chinese platform^1^ and conducted collectively by class. The class monitor sent the online survey link to the class group, and a graduate student in applied psychology served as the principal investigator. Instructions clarified the purpose of the survey and emphasized careful completion, voluntary participation, and confidentiality. After data cleaning, 335 valid questionnaires were obtained, yielding a valid response rate of 86.5%. Among them, 209 were female (62.4%). Participants were 18–25 years (M = 19.96, SD = 1.26). Regarding average daily SM use: 38 participants (11.3%) used it for less than 1 h; 78 (23.3%) used 1–2 h; 55 (16.4%) used 2–3 h; 48 (14.3%) used 3–4 h; 53 (15.8%) used 4–5 h; and 63 (18.8%) used more than 5 h. This indicates that more than one-third of the participants used social media for more than 4 h a day.

#### 2.1.2 Measurement tools

##### 2.1.2.1 Interdependent self-construal scale

The interdependent self-construal subscale of the Chinese version of the Self-Construal Scale (SCS), revised by Wang et al. (2008), which was developed by Singelis (1994). The interdependent self-construal subscale consists of 12 items on a 7-point Likert scale, with 1–7 representing “strongly disagree—strongly agree.” The higher the score, the higher the level of interdependent self-construal. In this study, the interdependent self-construal subscale Cronbach’*α* was 0.81, with good structural validity: (*χ^2^/df* = 2.79, CFI = 0.91, TLI = 0.89, SRMR = 0.06, RMSEA = 0.07).

##### 2.1.2.2 Need to belong scale

The need to belong scale, revised by Leary et al. (2013). The Chinese version of the scale is widely used in China. It has good reliability and validity (Mo et al., 2020), a total of 10 items, including 3 reverse items and 7 forward items, were scored using 7-point Likert scores, 1–7 representing “very inconsistent—very consistent,” and the reverse questions were added after the reverse score. The higher the score, the stronger the need for an individual to belong. In this study, the Cronbach’*α* of the need scale was 0.72, with good structural validity: (*χ^2^/df* = 2.81, CFI = 0.93, TLI = 0.90, SRMR = 0.06, RMSEA = 0.07).

##### 2.1.2.3 Trait–state FoMO scale

The Chinese trait–state FoMO Scale, translated and revised by Li et al. (2020), was compiled by Wegmann et al. (2017). The Chinese version of the scale consists of 12 items, based on the trait-FoMO (e.g., “I fear that other people have more wonderful experiences and gains than I do”). Moreover, state-FoMO (e.g., “I stay online all the time so I do not miss anything.”) The two-dimensional structure, including 5 items and 7 items, respectively, adopts a 5-point Likert scale, 1–5 means “completely disagree - completely agree,” and the higher the score, the higher the level of the individual’s FoMO. In this study, the Cronbach’α of the whole scale was 0.87, and the subscales were successively 0.80 and 0.83, all with good structural validity: (*χ^2^/df* = 4.10, CFI = 0.98, TLI = 0.95, SRMR = 0.06, RMSEA = 0.09), (*χ^2^/df* = 2.99, CFI = 0.97, TLI = 0.95, SRMR = 0.03, RMSEA = 0.07).

##### 2.1.2.4 Active social media use

The scale has eight questions, which were translated into Chinese and modified by Liu et al. (2017). Questions 1–4 in the “Surveillance use” scale (Tandoc et al., 2015) were used to measure active social media use. Moreover, the last 4 questions, about the level of passive social media use, were not adopted. It has good structural validity. The scale uses a 5-point Likert scale, with 1–5 representing “never—frequently.” The higher the score, the higher the level of active media use. In this study, the Cronbach’α of the active SM use subscale was 0.70, with good structural validity: (*χ^2^/df* = 023, CFI = 0.99, TLI = 0.99, SRMR = 0.00, RMSEA = 0.00).

##### 2.1.2.5 Control variables

Previous studies have shown that gender, age, and time spent on SM have impacted FoMO (Fioravanti et al., 2021; Przybylski et al., 2013; Xie et al., 2018; Zhang et al., 2021); therefore, this study collected these data for control during statistical analysis. Gender is represented by dummy variables “0” and “1,” and the SM use time report is the range (hours) of the average SM use time of the participants in the last week.

#### 2.1.3 Data analysis

The data were processed and analyzed using SPSS 26.0 and AMOS 23.0. Common method bias was assessed using Harman’s single-factor test. The results showed 12 factors with eigenvalues greater than 1, and the first factor explained 17.65% of the variance (<40%). Therefore, common method bias is not a serious concern, and subsequent analyses can proceed (Podsakoff et al., 2012). Construct validity was evaluated using confirmatory factor analysis. The relationships among key variables were investigated using Pearson correlation analysis. The moderated mediation model was examined using a bias-corrected nonparametric percentile bootstrap procedure with 5,000 resamples.

### 2.2 Results

#### 2.2.1 Descriptive statistics and correlation analysis

Table 1 shows the mean value of the control and research variables, as well as the standard deviation and correlation matrix. The results show that interdependent self-construal, need to belong, and active SNS use in Study 1 significantly correlate with trait-FoMO and state-FoMO.

**Table 1:** Descriptive statistics and correlation analysis of variables in Study 1 (*n*_1_ = 335) and Study 2 (*n*_2_ = 162).

Variable	1	2	3	4	5	6	7	8	9
1 Interdependent self-construal		0.24^**^	−0.04	−0.04	0.21^**^	−0.11	−0.07	−0.01	0.11
2 Need to belong	0.43^***^		−0.02	0.38^***^	0.34^***^	−0.13	−0.1	0.17^*^	−0.21^**^
3 Active SM use	0.16^**^	0.17^**^		−0.04	−0.03	0.12	−0.04	−0.01	0.02
4 Trait-FoMO	0.14^**^	0.36^***^	0.12^*^		0.53^***^	−0.04	−0.09	0.28^***^	−0.23^**^
5 State-FoMO	0.26^***^	0.28^***^	0.29^***^	0.56^**^		−0.02	−0.11	0.29^***^	0.02
6 Gender	−0.06	0.21^***^	0.10	0.08	−0.03		0.21^**^	−0.02	0.03
7 Age	−0.01	0.03	−0.08	−0.05	−0.05	−0.14^**^		−0.11	−0.15
8 Average SM use time	0.11^*^	0.16^**^	0.22^***^	0.08	0.11^*^	0.13^*^	0.01		−0.06
9 Emotional level	—	—	—	—	—	—	—	—	
*M_1_*	5.18	4.56	2.51	2.63	2.65	0.62	19.96	3.56	—
*SD_1_*	0.67	0.69	0.61	0.72	0.68	0.48	1.26	1.68	—
*M_2_*	0.52	4.85	0.51	2.87	2.61	0.72	20.48	4.76	6.82
*SD_2_*	0.5	0.77	0.5	0.77	0.74	0.45	1.94	3.00	2.03

The lower triangle represents the correlation coefficient of Study 1, and the upper triangle represents the correlation coefficient of Study 2. In Study 1, the time range was obtained, while in Study 2, the specific time was measured in hours. Gender is a dummy variable, with male = “0” and female = “1.” The levels of interdependent self-construal and active SM use in Study 2 are both dummy variables, with interdependent/high-level active SM use = “1” and independent/low-level active SM use = “0.” *p < 0.05, **p < 0.01, ***p < 0.001, same below.

#### 2.2.2 Test of the moderated mediation model

Model 14 in the SPSS macro program PROCESS, developed by Hayes (2017), was adopted. Controlling for gender, age, and average daily time spent on SM, we analyzed whether the mediating effect of need to belong between interdependent self-construal and trait/state-FoMO (the latter half) was moderated by active SM use, with all variables standardized.

To examine whether the mediating effect of need to belong between interdependent self-construal and trait-FoMO is moderated by active SM use. The results are shown in Table 2 (standardized). When the need to belong is not included in the regression equation, interdependent self-concept is an important predictive trait-FoMO (*β* = 0.14, *p* < 0.05). After inclusion of need to belong, interdependent self-construal significantly positively predicted need to belong (*β* = 0.43, *p* < 0.01), and need to belong was a significant positive predictor of trait-FoMO (*β* = 0.37, *p* < 0.01). The direct effect of interdependent self-construal on trait-FoMO was insignificant (*β* = −0.02, *p* = 0.73 > 0.05), indicating that the need to belong completely mediated the influence of interdependent self-construal on trait-FoMO. Finally, the interaction terms used by need to belong and active SM use had no significant predictive effect on trait-FoMO (*β* = −0.02, *p* = 0.71 > 0.05, 95% CI [−0.12, 0.08]).

**Table 2:** Moderated mediating effect test of interdependent self-construal on trait-FoMO (Study 1, n_1_ = 335).

Predictive variables	Model 1 (Trait-FoMO)			Model 2 (Need to belong)			Model 3 (Trait-FoMO)		
	*β*	*SE*	95% CI	*β*	*SE*	95% CI	*β*	*SE*	95% CI
Gender	0.07	0.05	[−0.03, 0.18]	0.22^***^	0.05	[0.13, 0.32]	−0.01	0.05	[−0.12, 0.09]
Age	−0.05	0.05	[−0.16, 0.05]	0.07	0.05	[−0.02, 0.16]	−0.08	0.05	[−0.18, 0.03]
SM use time	0.05	0.05	[−0.05, 0.16]	0.08	0.05	[−0.01, 0.17]	0.01	0.05	[−0.09, 0.12]
Interdependent self-construal	0.14^*^	0.05	[0.03, 0.25]	0.43^***^	0.05	[0.34, 0.53]	−0.02	0.06	[−0.14, 0.09]
Need to belong							0.37^***^	0.06	[0.25, 0.48]
Active SM use							0.05	0.05	[−0.05, 0.15]
Need to belong×Active SM use							−0.02	0.05	[−0.12, 0.08]
*R^2^*	0.03			0.25			0.14		
*F*	2.90^*^			27.74^***^			7.61^***^		

To examine whether the mediating effect of need to belong between interdependent self-construal and state-FoMO is moderated by active SM use. The results are shown in Table 3 (standardized). When need to belong was not included in the regression equation, interdependent self-construal significantly predicted state-FoMO (*β* = 0.25, *p* < 0.01). After inclusion of need to belong, interdependent self-construal significantly positively predicted need to belong (*β* = 0.43, *p* < 0.01), and need to belong significantly positively predicted state-FoMO (*β* = 0.37, *p* < 0.01, *β* = 0.19, *p* < 0.01). The direct effect of interdependent self-construal on state-FoMO was significant (*β* = 0.16, *p* < 0.01), indicating that the need to belong partially mediated the influence of interdependent self-construal on state-FoMO. The interaction terms between active SM use and need to belong predicted the state-FoMO significantly (*β* = −0.14, *p* < 0.01).

**Table 3:** Moderated mediating effect test of interdependent self-construal on state-FoMO (Study 1, n_1_ = 335).

Predictive variables	Model 1 (State-FoMO)			Model 2 (Need to belong)			Model 3 (State-FoMO)		
	*β*	*SE*	95% CI	*β*	*SE*	95% CI	*β*	*SE*	95% CI
Gender	−0.03	0.05	[−0.14, 0.07]	0.22^***^	0.05	[0.13, 0.32]	−0.11^*^	0.05	[−0.21, −0.01]
Age	−0.06	0.06	[−0.16, 0.05]	0.07	0.05	[−0.02, 0.16]	−0.05	0.05	[−0.15, 0.05]
SM use time	0.09	0.05	[−0.02, 0.19]	0.08	0.05	[−0.01, 0.17]	0.02	0.05	[−0.08, 0.12]
Interdependent self-construal	0.25^**^	0.05	[0.15, 0.36]	0.43^***^	0.05	[0.34, 0.53]	0.13^*^	0.06	[0.02, 0.2]
Need to belong							0.19^**^	0.06	[0.07, 0.30]
Active SM use							0.24^***^	0.05	[0.13, 0.34]
need to belong×active SNS use							−0.14^**^	0.05	[−0.24, −0.04]
*R^2^*	0.08			0.25			0.19		
*F*	7.27^***^			27.74^***^			10.82^***^		

**Figure 2:**
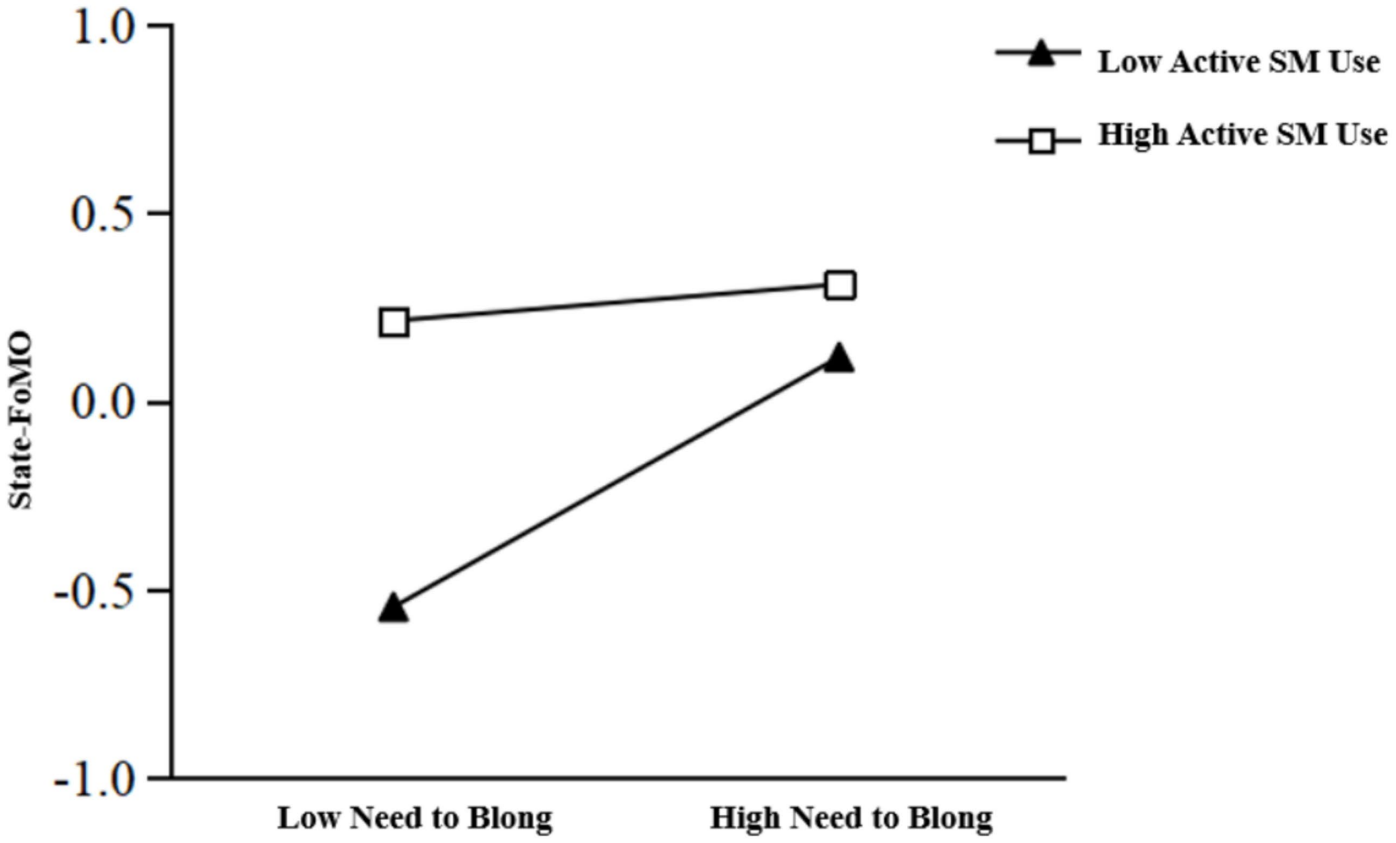
The moderating effect of active SM use on the relationship between need to belong and state-FoMO (Study 1).

The simple slope was used to analyze the moderating effect of active SM use. As shown in Figure 2, among individuals with low level (one standard deviation below the mean) of active SM use, need to belong had a significant positive predictive effect on state-FoMO (*β* = 0.33, *p* < 0.01, 95% CI [0.18, 0.47]). The prediction effect was not significant in individuals with high levels (one standard deviation above the mean) (*β* = 0.05, *p* = 0.53 > 0.05, 95% CI [−0.10, 0.20]). In addition, Table 4 shows that, from the low level of active SM use to the high level, the mediating effect of need to belong decreases in the relationship between interdependent self-construal and state-FoMO.

**Table 4:** Mediating effects of need to belong under high and low levels of active SM use.

Study	Active SM use	Indirect effect	Boot *SE*	95% CI low	95% CI up
Study 1	High	0.02	0.04	−0.05	0.11
	Low	0.14	0.03	0.08	0.21
Study 2	High	0.02	0.03	−0.03	0.08
	Low	0.13	0.04	0.05	0.22

## 3 Study 2: situational simulation experiment

The primary purpose of Study 2 is to validate the findings obtained in Study 1. On one hand, manipulating self-construal allows for further testing of Hypotheses 1 and 2, providing causal evidence for the effect of interdependent self-construal on need to belong and trait/state-FoMO. On the other hand, by changing the measurement of active SM use from self-report to situational manipulation, Hypothesis 3 is further tested, examining the robustness of the moderating effect found in Study 1.

### 3.1 Methods

#### 3.1.1 Participants

A total of 190 university students from a university in Nanchang city, China, each with over 1 year of experience using SM (QQ, WeChat Moments, etc.) and who had not participated in Study 1, were recruited online. All participants were randomly assigned to different conditions to complete an online questionnaire via the Wenjuanxing platform, with compensation provided upon completion. After removing invalid questionnaires (3 participants who self-reported careless responses and 25 with excessively long or short completion times; see the supplementary note ^2^), 162 valid responses remained, including 46 males and 116 females, aged 18–25 years (M = 20.48, SD = 1.91). The average daily SM use was 4.76 h.

#### 3.1.2 Materials

##### 3.1.2.1 Self-construal manipulation

Referring to the research of Su (2017) and Huang (2020), the “story priming method” combined with the “pronoun circle method” is adopted. The story priming method adopts the paradigm of Trafimow et al. (1991), requiring participants to read a short story about Sumerian soldiers. The first part of the story, read by the interdependent and independent groups, is the same. The information read by the interdependent group highlights the family, while the information read by the independent group highlights the individual. Using the paradigm of Stapel and Koomen (2001), participants were asked to read an essay describing a city trip. The two groups had duplicate content. The difference was that all the essays contained “we” and “our” in the interdependent group, while all the pronouns were “me” and “mine” in the independent group. Since this study was conducted online, the task was changed to ask participants to pay special attention to all pronouns when reading the text. In this study, all participants must read two short essays and answer two manipulation test questions. Question 1 is “To what extent do you feel relevant to others at this moment (positive score),” using a 7-point scale, “1 = completely irrelevant, 7 = completely related “. Question 2 is “To what extent do you feel separate from others at this moment (reverse scoring),” using a 7-point scale, “1 = not separated at all, 7 = completely separated.” The two scores are added together; the higher the score, the more relevant the self is to others. Please refer to the Supplementary materials for detailed information on the experimental materials.

##### 3.1.2.2 Active SM use manipulation

This study is based on the definition, scale, and related literature of active SM use (Verduyn et al., 2015; Tandoc et al., 2015; Verduyn et al., 2017), and compiled the manipulative materials, which were divided into two different scenarios of high level and low level. The first part of the two groups of materials is the same, which roughly describes that SM is a popular tool that we use every day. For example, we can use it to communicate and browse the news, etc. Then, the participants were told to imagine and recall according to the requirements of the following time, regardless of how they had used SM before. The second half is different: the high-level group participants were asked to imagine that they were a user who often used SNS to interact directly with their friends (such as commenting on the news, actively sending messages to friends, etc.), and were asked to recall the last time they truly participated in direct communication activities, while reporting the specific contents of the activities as detailed as possible. In the lower group, participants were asked to imagine themselves as users who rarely interact directly with others on SM. Please refer to the Supplementary materials for detailed information on the experimental materials.

In the preliminary experiment, 32 university students who did not participate in the formal experiment evaluated the materials. After reading the situational materials and descriptions, they were asked to answer, “I often have direct communication with my friends or cyber friends on SM,” with a 7-level rating of “1 = completely disagree −7 = completely agree.” High-level group (*n* = 16, M = 5.06, SD = 1.06) and the low-level group (*n* = 16, M = 3.75, SD = 1.46) showed significant differences (t (30) = 2.94, *p* < 0.01, Cohen’s d = 1.04). Therefore, the situational material can manipulate both high and low levels of active SM use and can be used in formal experiments.

##### 3.1.2.3 Need to belong scale, trait–state FoMO scale

In study 2, the Need to belong scale reliability was 0.75, and the structural validity was (*χ^2^/df* = 1.98, CFI = 0.92, TLI = 0.88, SRMR = 0.06, RMSEA = 0.07). The reliability and structural validity of the trait-FoMO subscale were 0.80 (*χ^2^/df* = 1.41, CFI = 0.99, TLI = 0.98, SRMR = 0.03, RMSEA = 0.05). The reliability and structural validity of the state-FoMO subscale were 0.86 (χ^2^/df = 1.81, CFI = 0.97, TLI = 0.96, SRMR = 0.04, RMSEA = 0.07).

##### 3.1.2.4 Control variables

First of all, the randomization of the online platform ensures the homogeneity of the participants to a large extent. It avoids the baseline differences in the main variables of different groups, so there is no need to measure and screen the main variables of the baseline. Secondly, FoMO, as a subtype of anxiety, is affected by emotions (Elhai et al., 2020a). Therefore, the baseline emotional level of the subjects was measured before the experiment, and it was used as one of the control variables. The mood at that time was rated “1-very bad, 10-very good.” Finally, as in study 1, gender, age, and time spent on SM were also used as control variables.

#### 3.1.3 Procedure

First, all participants read the guidance and informed consent on the front page of the online questionnaire, and assessed their emotional level before the experiment. Second, the participants were randomly assigned to one of two conditions (interdependent self-construal group and independent self-construal group). In order to ensure the quality of the experiment, the minimum answering time was set for each condition according to the normal reading speed of the material, and the next step could not be entered until the time was up. After completing the self-construal priming, all participants completed the need to belong scale. Third, the subjects in the two self-construal groups were randomly assigned to one of the high/low level active SM groups, comprising four groups. They also set a minimum time to answer the question, in which the recall session of the high-level group was not less than 2 min. After completing active SM use level manipulation, all participants completed the trait–state FoMO scale. Finally, all subjects filled in the gender, age, average time (hours) per day of SM use, and whether they were careful to fill in the self-assessment (self-rated not serious answer subjects were excluded).

#### 3.1.4 Data analysis

Building on the methods used in Study 1, Study 2 additionally employed independent-samples *t*-tests and analysis of variance (ANOVA) to examine group differences.

### 3.2 Results

#### 3.2.1 Manipulation test

The results of the independent sample T-test showed that the scores of interdependent self-construal (*M* = 4.41, *SD* = 0.85) and independent self-construal (*M* = 3.98, *SD* = 1.06) were significantly different (*t* = 2.83, *p* < 0.01, Cohen’s *d* = 0.45), and the manipulation of different self-construal was successful. The scores of the group with high (*M* = 4.68, *SD* = 1.41) and low (*M* = 3.76, *SD* = 1.68) active SM use were significantly different (*t* = 3.77, *p* < 0.001, Cohen’s *d* = 0.59); the level of active SM use was successfully manipulated.

#### 3.2.2 Descriptive statistics and correlation analysis

The data in the upper triangle of Table 1 are shown. In addition to no relationship between interdependent self-construal and trait-FoMO, the relationship between other major variables was significant, consistent with Study 1.

#### 3.2.3 Test of the moderated mediation model

According to the suggestions of Wen and Ye (2014), the mediation analysis of independent variables as binary category variables, mediations, and dependent variables as continuous variables is consistent with the analysis of continuous variables. For specific operations, refer to the description of study 1. It should be added that in the self-construal, the interdependent group is coded as “1” and the independent group is “0.” The emotion level is also added to the control variable. The results showed (standardized) that when need to belong was not included in the regression equation, interdependent self-construal could not significantly predict trait-FoMO (*β* = 0.06, *p* = 0.45 > 0.05, 95% CI [−0.08, 0.21]), but was able to significantly positively predict state-FoMO (*β* = 0.21, *p* < 0.01, 95% CI [0.05, 0.35]). After adding need to belong, interdependent self-construal significantly positively predicted need to belong (*β* = 0.24, *p* < 0.01, 95% CI [0.11, 0.39]). At the same time, need to belong significantly positively predicts trait-FoMO (*β* = 0.31, *p* < 0.001, 95% CI [0.16, 0.46]) and state-FoMO (*β* = 0.29, *p* < 0.001, 95% CI [0.14, 0.45]). In contrast, the direct effect of interdependent self-construal on trait-FoMO is not significant (*β* = 0.01, *p* = 0.84 > 0.05, 95% CI [0.16, 0.14]), and on state-FoMO is significantly marginal (*β* = 0.12, *p* = 0.08, 95% CI [0.01, 0.26]). The interaction term between active SM and the need to belong does not significantly predict trait-FoMO (*β* = 0.02, *p* = 0.71 > 0.05, 95% CI [0.17, 0.11]), but is significant for the forecast of state-FoMO (*β* = 0.23, *p* < 0.01, 95% CI [0.36, 0.08]).

**Figure 3:**
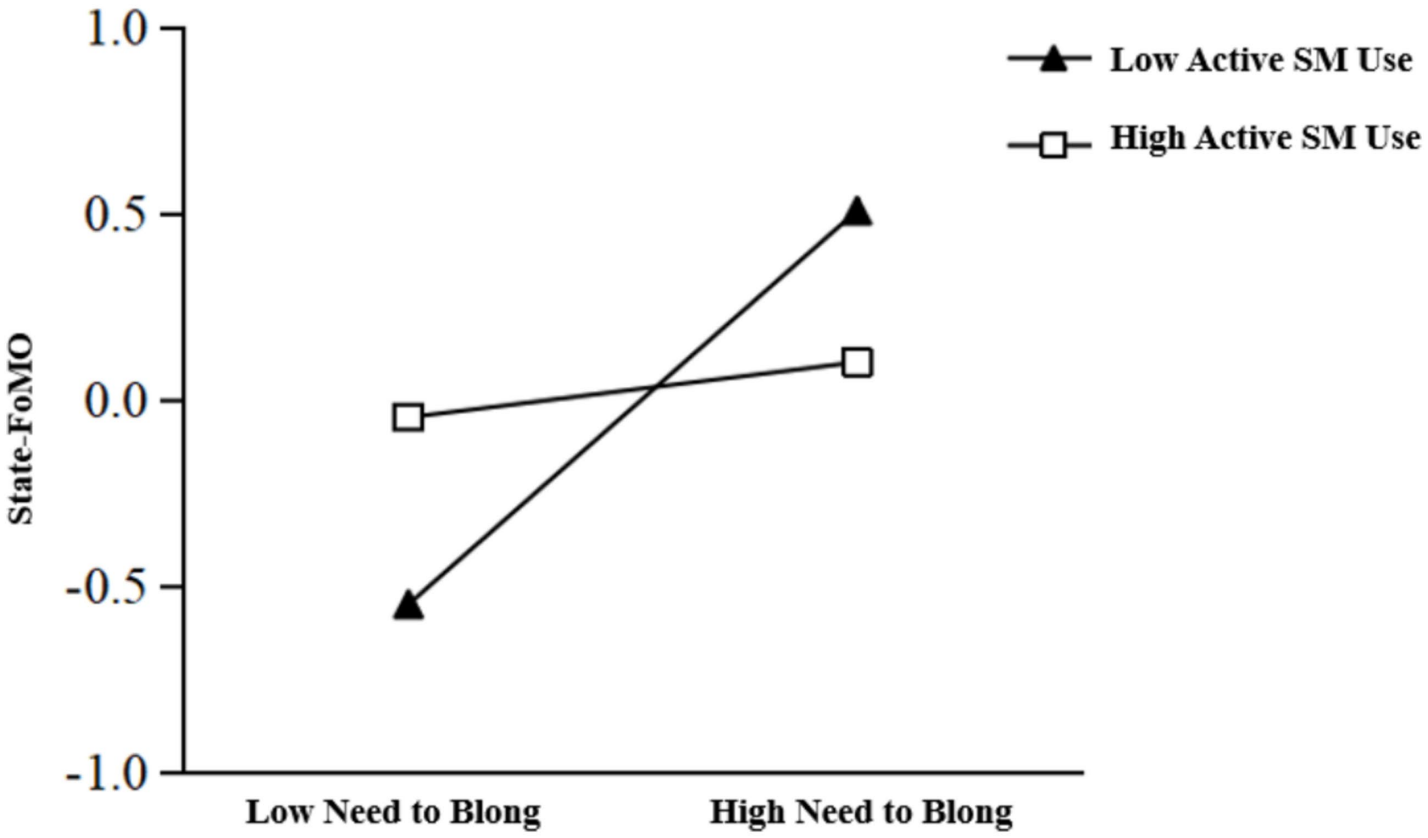
The moderating effect of active SM use on the relationship between need to belong and state-FoMO (Study 2).

**Figure 4:**
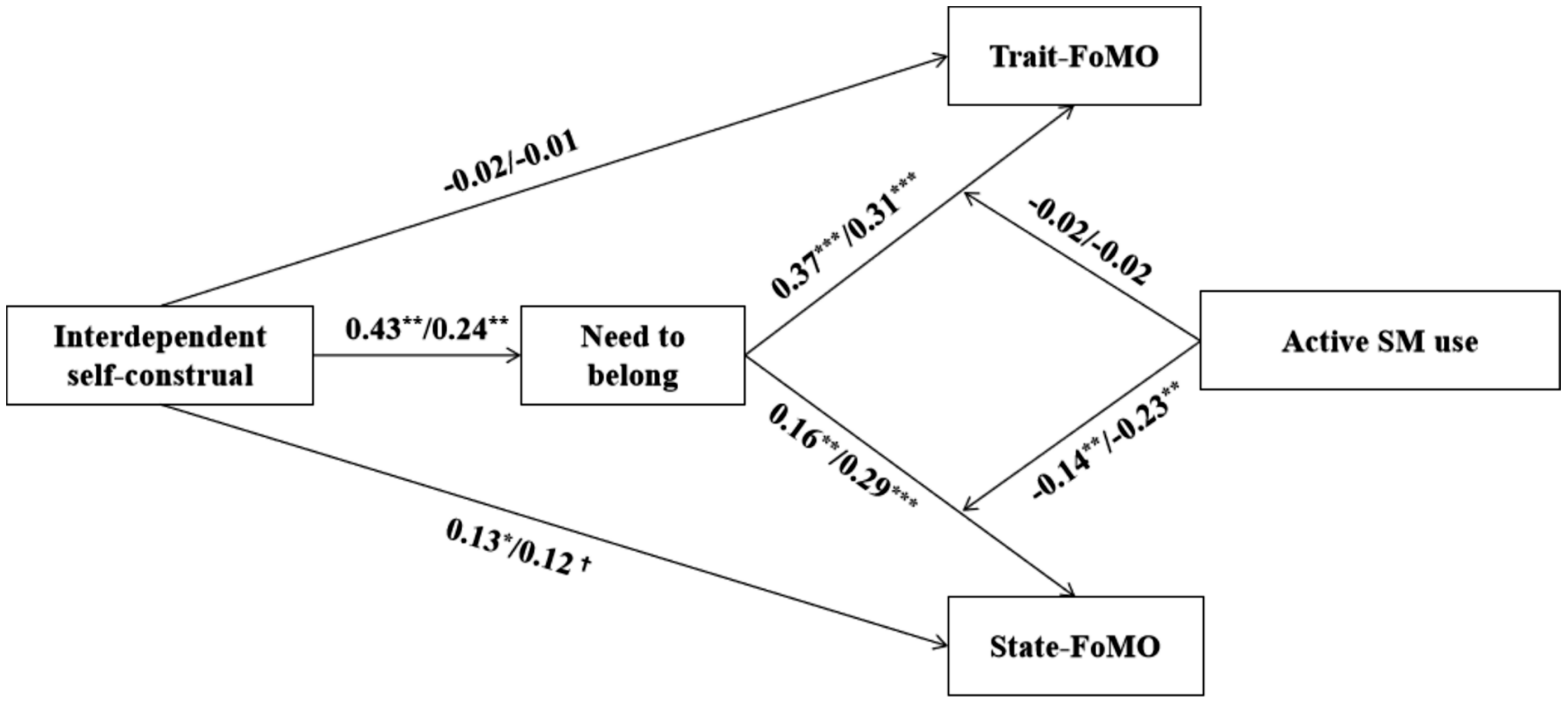
Path results of study 1 and study 2. *n_1_* = 335, *n_2_* = 162. For the sake of simplicity, the control variables are not indicated. The coefficients in the figure are standardized coefficients, with the coefficients on the left representing the results from Study 1 and on the right from Study 2. ^†^*p* < 0.1.

A simple slope was used to analyze the moderating effect of active SNS use on the relationship between the need to belong and state-FoMO. As shown in Figure 3, in the group with low-level active SNS use, the need to belong had a significant positive predictive effect on state-FoMO (*β* = 0.52, *p* < 0.001, 95% CI [0.32, 0.74]). Forecast effect is not significant in the high level group (*β* = 0.06, *p* = 0.46 > 0.05, 95% CI [0.13, 0.27]). The mediating effects of need to belong in the high and low level groups are shown in Table 4. Figure 4 shows the path results (standardized) of the research model from Studies 1 and 2.

#### 3.2.4 Robustness test of trait/state-FoMO difference after initiating self-construal

In order to test whether the manipulation of active SNS use level affects the differences in trait/state-FoMO level after different self-construal initiation, a two-factor analysis of variance was used to evaluate the effects. The results showed that the main effect of self-construal and active SM use on trait-FoMO was not significant (*F*(1,158) = 0.35, *p* = 0.55 > 0.05; *F*(1,158) = 0.42, *p* = 0.52 > 0.05), the interaction items in self-construal and active SM also had no significant influence on trait-FoMO (*F*(1,158) = 1.08, *p* = 0.29 > 0.05). The main effect of self-construal on the state-FoMO was significant (*F*(1,158) = 7.07, *p* < 0.01, η^2^ = 0.05), while the main effect of active SM use on state-FoMO was not significant (*F*(1,158) = 0.09, *p* = 0.76 > 0.05). The interaction items in self-construal and active SM also had no significant influence on state-FoMO (*F*(1,158) = 0.001, *p* = 0.97 > 0.05). In this study, the difference in state-FoMO under different self-construal primes was not affected by the level of active SNS use manipulation.

## 4 Discussion

This study focused on Chinese university students and conducted two studies to examine the effects of interdependent self-construal on trait and state-FoMO. It revealed the significant mediating role of the need to belong and confirmed the moderating effect of active SM use on the relationship between need to belong and state-FoMO. From a multi-factor integrative perspective, this study carefully distinguished between the intensity and satisfaction of psychological needs, effectively integrating psychological factors (need to belong) and behavioral factors (active SM use). It deepened and extended the understanding of the mechanisms through which self-construal influences FoMO and identified differences in these mechanisms between trait and state-FoMO.

### 4.1 Interdependent self-construal and trait- versus state-FoMO

The findings suggest that interdependent self-construal may relate differently to trait-FoMO and state-FoMO. In Study 1, interdependent self-construal was positively correlated with trait-FoMO, but this association became non-significant after need to belong was included in the model. In Study 2, the short-term activation of interdependent self-construal did not significantly change trait-FoMO. These results suggest that interdependent self-construal may not be a robust direct correlate of trait-FoMO among Chinese university students. Rather, its association with trait-FoMO appears to operate mainly through belongingness needs.

This pattern differs from Dogan’s (2019) findings. Dogan (2019) reported a stronger association between interdependent self-construal and FoMO and found that priming interdependent self-construal increased FoMO among American participants. In the present study, the association between dispositional interdependent self-construal and trait-FoMO was weaker and was closer to findings from another Chinese sample (Li et al., 2023). One possible explanation concerns the cultural context. In collectivistic cultural settings such as China, interdependent self-construal is more consistent with dominant cultural norms emphasizing relational harmony and social connectedness (Markus and Kitayama, 1991; Morling, 2016). Under such conditions, interdependence may support social integration and psychological adjustment, rather than necessarily intensifying generalized concerns about missing out (Hu et al., 2012; Qiao and Wu, 2021; Zhang, 2012). By contrast, in more individualistic cultural contexts, stronger interdependent self-construal may be less congruent with dominant cultural values and may be more closely associated with interpersonal concerns or negative affect (Maas et al., 2019).

However, this cultural explanation is speculative. The present study did not include a direct cross-cultural comparison. Nor did it test whether cultural values moderated the association between interdependent self-construal and FoMO. Therefore, the current results cannot demonstrate that culture explains the difference between the present findings and those of Dogan (2019). Future research should use multicultural samples and measurement-invariant designs to directly examine whether cultural context shapes the relationship between self-construal and FoMO.

The pattern was more consistent for state-FoMO. In both studies, interdependent self-construal was more directly associated with state-FoMO than with trait-FoMO. This is consistent with the trait–state FoMO framework. State-FoMO is more situation-sensitive and more closely tied to online contexts (Wegmann et al., 2017; Röttinger et al., 2021). Interdependent self-construal emphasizes relational connectedness and attention to others. When this orientation is activated, individuals may become more sensitive to social information, interpersonal availability, and possible disconnection from online interactions. This may explain why interdependent self-construal showed a more direct association with state-FoMO. Nevertheless, the observed effects were modest, and further evidence is needed before drawing strong conclusions.

### 4.2 The mediating role of need to belong

Need to belong appeared to be an important psychological pathway linking interdependent self-construal to both forms of FoMO. Due to their greater emphasis on interpersonal relationships, individuals with high interdependent self-construal have stronger needs for belonging. The need to belong, a basic motivation for establishing and sustaining interpersonal relationships, prompts individuals to actively maintain positive social ties (Baumeister and Leary, 1995), heightening their attention to others and fostering worry or anxiety about a lack of connection (Beyens et al., 2016; Wang et al., 2018). Consequently, those with high social needs are more prone to concern that they might miss out on valuable experiences of others.

For trait-FoMO, the indirect association through the need to belong was the primary pathway observed in the present study. This finding is consistent with the view that trait-FoMO reflects a relatively stable and generalized concern about missing rewarding social experiences (Przybylski et al., 2013; Wegmann et al., 2017). Trait-FoMO may therefore be closely tied to enduring motivational tendencies, such as a strong need for belonging and social connection (Röttinger et al., 2021).

For state-FoMO, interdependent self-construal showed both a direct association and an indirect association through the need to belong. The findings further underscore the situational instability of state-FoMO, allowing it to be affected by both distal and proximal factors. Moreover, state-FoMO is more closely related to SM use (Wegmann et al., 2017; Röttinger et al., 2021). Individuals with high interdependent self-construal and a high need to belong, due to their focus on interpersonal relationships, tend to engage in greater SM use (Chen and Marcus, 2012; Chang, 2015). Thus, interdependent self-construal may be linked to state-FoMO by strengthening belongingness needs and by increasing sensitivity to online social cues. However, this pathway should not be interpreted as causal evidence. Study 1 was cross-sectional. In Study 2, need to belong and FoMO were still assessed through self-report. Longitudinal and experimental studies are needed to clarify whether belongingness needs causally link interdependent self-construal to FoMO.

### 4.3 Active social media use as a boundary condition

A central finding was that active SM use attenuated the positive association between need to belong and state-FoMO. This pattern did not emerge for trait-FoMO. As noted in the introduction, individuals with high belongingness needs exhibit strong motivations for social connection and a keen desire to track others’ activities (Baumeister and Leary, 1995; Wang et al., 2018). Active SM use can satisfy these belongingness needs (Baumeister and Leary, 1995; Malik et al., 2016), thereby maintaining better self-regulation and more positive emotional experiences (Ryan and Deci, 2000; Przybylski et al., 2013). When individuals with high belongingness needs successfully experience connection and recognition through active SM interactions, their belongingness needs are partially “satisfied” or “buffered.” This sense of fulfillment can effectively reduce anxiety arising from the fear of social exclusion, i.e., FoMO. Under these circumstances, strong belongingness needs are less likely to manifest as negative FoMO. This interpretation is consistent with recent work on the social-bonding basis of FoMO. Rifkin et al. (2025) showed that FoMO is intensified when individuals perceive that they have missed social bonding with valued groups. They also found that recalling previous social connection can temporarily reduce FoMO. Similarly, active online interaction may provide immediate social feedback and affirm that one remains connected to others. This may be especially relevant for state-FoMO, which is online-specific and situation-sensitive.

At the same time, the current findings should not be interpreted as showing that active SM use generally reduces FoMO. Active SM use may have both positive and negative implications. It may enhance social connection and interpersonal satisfaction, but it may also increase exposure to others’ activities and thereby intensify concerns about missing out (Mao et al., 2023; Mao and Zhang, 2023). Thus, the current result is best understood as a conditional pattern. Among individuals with stronger belongingness needs, active SM use may be associated with weaker state-FoMO when it provides social feedback and momentary connectedness.

The absence of a significant moderating effect for trait-FoMO is also meaningful. Trait-FoMO reflects a more stable and generalized tendency to worry about missing rewarding experiences (Wegmann et al., 2017; Röttinger et al., 2021). Temporary online connection may not be sufficient to change this generalized tendency. In addition, online social connection can be unstable or incomplete and may not fully satisfy deeper belongingness needs (Kraut et al., 1998; Newson et al., 2021). Additionally, increased SM use can displace offline activities, potentially heightening concerns about missing important real-life events, thus exacerbating FoMO (Burnell et al., 2019; Zhang et al., 2021). This may explain why active SM use buffered state-FoMO but not trait-FoMO.

### 4.4 Research significance and implications

First, it adds to the trait–state FoMO literature by showing that interdependent self-construal, need to belong, and active SM use may operate differently across the two forms of FoMO. Trait-FoMO was mainly linked to interdependent self-construal through need to belong, whereas state-FoMO showed a more direct association with interdependent self-construal and was more sensitive to active SM use. This supports the value of distinguishing generalized FoMO tendencies from online-specific momentary FoMO experiences. Second, the study extends previous work on self-construal and FoMO by identifying need to belong as a plausible psychological pathway, especially for trait-FoMO (Dogan, 2019). Third, the findings suggest that active SM use should not be viewed as uniformly harmful or beneficial. Its role may depend on the type of FoMO and the individual’s belongingness needs. Active online interaction may provide immediate social feedback and connection, thereby weakening the association between the need to belong and state-FoMO, but such temporary connection may be insufficient to alter trait-FoMO.

The practical implications should also be interpreted cautiously. The findings do not imply that universities should simply encourage more SM use among students. Instead, interventions aimed at FoMO may benefit from focusing on the quality of social connection and the satisfaction of belongingness needs. Universities could provide structured opportunities for students to build offline belonging. Examples include peer mentoring, small-group activities for first-year students, class-based community building, and counseling workshops on interpersonal connection and social comparison. In online contexts, students could be guided to engage in more supportive and interactive forms of SM use, such as direct communication, constructive commenting, help-seeking, and authentic self-disclosure. These suggestions are preliminary and should be tested in intervention studies.

### 4.5 Research limitations and future directions

There are still several limitations in the current study. First, causal conclusions remain limited. Study 1 was cross-sectional, and the mediation and moderation patterns cannot establish temporal order. Study 2 used experimental manipulations, but the mediator and outcomes were still measured through self-report. Future longitudinal, experimental, and ecological momentary assessment studies are needed.

Second, both studies relied heavily on self-report measures, which may introduce shared method variance, social desirability, and subjective response biases. Future studies should combine self-report scales with behavioral indicators, digital trace data, real-time SM interaction tasks, or peer reports. Third, several effects were small to modest, and some measures showed only modest internal consistency. Thus, the findings should be interpreted cautiously. Future research should use larger samples and refined measures to assess the stability and practical significance of these effects.

Fourth, the generalizability of the findings is limited because both samples consisted of Chinese university students. Future studies should test the model in adolescents, older adults, non-student populations, and other cultural contexts. Finally, the ecological validity of the Study 2 active SM use manipulation was limited. The task involved imagined and recalled online interaction rather than real-time SM behavior. It may have activated the psychological experience of online social connectedness rather than directly manipulating active SM use itself. Future research should use real-time online interaction tasks, platform-based behavioral logs, or experience sampling methods.

## 5 Conclusion

Grounded in the trait–state FoMO framework, this study examined the roles of need to belong and active SM use in the association between interdependent self-construal and FoMO. In two studies, interdependent self-construal was indirectly associated with trait-FoMO through the need to belong, whereas it showed both direct and indirect associations with state-FoMO. Active SM use attenuated the association between need to belong and state-FoMO, but not trait-FoMO. These findings suggest that trait- and state-FoMO may involve partly different psychological and behavioral processes. They also highlight the potential value of satisfying belongingness needs and promoting supportive active online interaction, while the findings should be interpreted cautiously due to the cross-sectional, self-report, and scenario-based features of the studies.

## Data Availability

The original contributions presented in the study are included in the article/Supplementary material, further inquiries can be directed to the corresponding author.

## Footnotes

1
www.wjx.cn

2 **Supplementary Notes:** First, six participants with experimental durations exceeding 20 min were excluded. Since the high-level active SM use initiation group required participants to recall information, while the low-level usage group did not, the average experimental times for the two groups should be calculated separately. Results showed the high-level group’s experimental time ranged from 266 to 966 s, with M = 483, SD = 157; the low-level group ranged from 193 to 743 s, with M = 374, SD = 113. Consequently, 19 participants were ultimately excluded: those in the high-level group with response times below 320 s and those in the low-level group with response times below 260 s.
