# Supplementary material for: From self-construal to trait- and state-fear of missing out: the roles of need to belong and active social media use
Source: Front Psychol. 2026 Jul 23;17:1743687. doi: 10.3389/fpsyg.2026.1743687 (PMC13445959; doi:10.3389/fpsyg.2026.1743687)
Supplement: Supplementary file 1 [file Supplementary_File_1.docx]

# Supplementary Materials: Situational Simulation Materials

## Self-Construction Manipulation：

1. **Interdependent self-construal group**
(1) **Read the following passage carefully, then select the answer you believe is most appropriate from the options provided.**

Sostratis was a warrior of the ancient Sumerian kingdom. He had rendered immortal service to Sargon I by helping him conquer the entire Mesopotamian region. To reward him, Sargon I granted him a small kingdom to rule independently. About ten years later, Sargon I faced a new war. Sostratis was obliged to send a soldier to assist him. But whom should he send? After much consideration, Sostratis finally decided to send Tego, a warrior from his own family.

This decision carried several advantages: first, Sostratis demonstrated loyalty to his family while also strengthening their loyalty toward him. Second, appointing Tego as commander would enhance the power and prestige of the whole family. Third, if Tego performed well, it would earn Sargon I’s gratitude toward Sostratis’s family.

Do you admire Sostratis’s wisdom?

①Yes. ②No. ③Uncertain

(2) **Read the passage, paying attention to all instances of the pronouns “we” or “our.”**

We often go to the countryside together. The rural scenery is like an expressive landscape painting, bringing a deep sense of peace to our hearts. Walking along the country paths, our eyes are filled with vibrant green, and occasionally, some unknown little flowers catch our attention, adding to our joy. Sometimes, we pick these flowers and weave them into crowns to wear on our heads. Other times, we quietly lie on the lush green grass, watching the white clouds drift across the blue sky. The vast sky and floating clouds spark our endless imagination.

At times, we run freely through the fields, flying our kites and letting our dreams soar. When we come to the countryside—when we pass over small bridges with flowing water, lie in the fields watching clouds, or run flying kites—we feel that we, too, have become strokes in that expressive landscape painting.

2. **Dependent self-construal group**
(1) **Read the following passage carefully, then select the answer you believe is most appropriate from the options provided.**

Sostratis was a warrior of the ancient Sumerian kingdom. He had rendered immortal service to Sargon I by helping him conquer the entire Mesopotamian region. To reward him, Sargon I granted him a small kingdom to rule independently. About ten years later, Sargon I faced a new war. Sostratis was obliged to send a soldier to assist him. But whom should he send? After much consideration, Sostratis finally decided to send Tego, his most capable general.

This decision carried several advantages. First, Sostratis could earn Tego’s gratitude, which would help him consolidate his territory. Second, sending such a talented general to fight would greatly enhance his reputation. Third, by dispatching his best general, Sostratis could gain Sargon I’s gratitude. Finally, this might also lead to new rewards from Sargon I.

Do you admire Sostratis’s wisdom?

①Yes. ②No. ③Uncertain.

**(2)Read the passage, paying attention to all instances of the pronouns “I” or “my.”**

I often go to the countryside alone. The rural scenery is like an expressive landscape painting, bringing a deep sense of peace to my heart. Walking along the country paths, my eyes are filled with vibrant green, and occasionally, some unknown little flowers catch my gaze, adding to my joy. Sometimes, I pick these flowers and weave them into crowns to wear on my head. Other times, I lie quietly on the lush green grass, watching the fluffy white clouds drift across the blue sky. The vast sky and floating clouds spark my endless imagination.

At times, I also run through the fields, flying my kite and letting my dreams soar. When I come to the countryside—when I pass over small bridges with flowing water, lie in the fields watching clouds, or run flying kites—I feel that I, too, have become a stroke in that expressive landscape painting.

***Manipulation Test***

①“To what extent do you feel connected to others at this moment?” “1 = Not at all connected — 7 = Completely connected.”

②“To what extent do you feel separated from others at this moment?” “1 = Not separated at all — 7 = Completely separated.”

## Level of Active Social Media Usage Manipulation

**1.High-Level Active Social Media Use Group**

Every day, more or less, we all use social media (such as QQ/QQ Zone, WeChat/Moments). Through them, we can communicate directly with others, update our own status, or browse others’ posts. Regardless of how you have used social media in the past, please imagine the following scenario:

In your upcoming social media use, you will frequently initiate chats with friends, actively comment on friends’ posts instead of just browsing them, update your own status, upload photos, and share things with friends. In short, you will engage in many activities that involve direct interaction with your friends while using social media.

Next, please recall the most recent time you engaged in these types of social media activities, and try to describe it in as much detail as possible. For example, were you actively chatting with friends or with family? What kind of status did you update? Whose posts did you comment on? How were you feeling at the time? Or any other details you think are relevant. In short, please try to describe as much as you can.

**2.Low-Level Active Social Media Use Group**

Every day, more or less, we all use social networks (such as QQ/QQ Zone, WeChat/Moments). Through them, we can communicate directly with others, update our status, or browse others’ posts. Regardless of how you have used social networks in the past, please imagine the following scenario:

In your upcoming social network use, you will rarely initiate chats with friends, seldom comment on friends’ posts, and mostly browse them. You rarely update your status, upload photos, or share things with friends. In short,you rarely engage in direct communication with friends while using social media.

***Manipulation Test***

Keep imagining, and evaluate the following statement.

①“I will frequently engage in direct communication with friends on social media platforms,“ ”1=Strongly disagree—7=Strongly agree."

**The following is the Chinese version**

## 自我建构操纵

### 1. 依存型自我建构组

（１）读短文，然后选出文后你认为最合适的答案。

索斯特瑞斯是古代苏美尔王国的一名勇士。他曾经为撒根一世征服整个美索不达米亚地区立下了不朽的战功。撤根一世为了奖励他，封给他一个小王国由他独自统治。大约又过了 10 年，撤根一世面临一场新的战争。索斯特瑞斯有义务派一名士兵去协助他。派谁去呢？索斯特瑞斯考虑了很久，最后决定派特各，一名家族成员中的勇士去。这个决定有许多好处：首先，索斯特瑞表现出了对家族的忠诚，也能巩固其他家族成员对自己的忠诚。其次，让特各当指挥官可以提高整个家族的权势和威望。第三，如果特各表现出色， 可以让撒根一世对自己的家族感恩。

#### 你欣赏索斯特瑞斯的睿智吗？

①是的②不是③不确定

（２）读短文，并注意文中出现的所有代词“我们”或“我们的”。

我们常一块去乡村玩。乡村的风景有如写意的山水画，会让我们的心变得十分宁静。走在乡间的小路上， 我们的眼里尽是生机勃勃的绿，偶尔有不知名的小花闯入我们的眼睑，增添我们的欢喜。有时候，我们会采摘一些，编成花冠戴在我们的头上。有时候，我们静静地躺在绿油油的草地上，看蓝蓝的天上朵朵白云飘荡。高远的天空，飘忽的白云，给我们无限遐想；有时候，我们也在田野里奔跑，放飞我们的风筝，也放飞我们的梦想。当我们来到乡村，当我们路过小桥流水，当我们在田野里躺在看白云，或者跑着放风筝， 我们想，我们一定也是那写意山水的一道道笔墨了。

### 2. 独立型自我建构组

（１）读短文，然后选出文后你认为最合适的答案。

索斯特瑞斯是古代苏美尔王国的一名勇士。他曾经为撒根一世征服整个美索不达米亚地区立下了不朽的战功。撤根一世为了奖励他，封给他一个小王国由他独自统治。大约又过了 10 年，撒根一世面临一场新的战争。索斯特瑞斯有义务派一名士兵去协助他。派谁去呢？索斯特瑞斯考虑了很久，最后决定派特各，一名最优秀的将军去。这个决定有很多好处：首先，索斯特瑞斯能够让最优秀的将军特各对自己感恩，从而帮助自己巩固领土。其次，让特各这样有才能的将军去征战，可以极大地提高自己的声誉。第三，派最能千的将军去帮忙，可以让撒根一世对自己感恩。最后，还可能从撤根一世那里得到新的奖赏。

#### 你欣赏索斯特瑞斯的睿智吗？

①是②不是③不确定

（２）认真阅读以下短文，并注意文中出现的所有代词“我”或“我的”。

我常独自去乡村玩。乡村的风景有如写意的山水画，会让我的心变得十分宁静。走在乡间的小路上，我的

眼里尽是生机勃勃的绿，偶尔有不知名的小花闯入我的眼睑，增添我的欢喜。有时候，我会采摘一些，编成花冠戴在我的头上。有时候，我静静地躺在绿油油的草地上，看蓝蓝的天上朵朵自云飘荡。高远的天空， 飘忽的白云，给我无限遐想；有时候，我也在田野里奔跑，放飞我的风筝，也放飞我的梦想。当我来到乡村，当我路过小桥流水，当我在田野里躺在看白云，或者跑着放风筝，我想，我一定也是那写意山水的一道笔墨了吧！

#### 操纵检验：

①“此刻你在多大程度上感觉自己是和他人相关的”，“1=完全不相关—7=完全相关”

②“此刻你在多大程度上感到自己和他人是分离的”，“1=完全不分离—7=完全分离”

## 主动性社交媒体使用水平操纵

### 1.主动性社交媒体使用高水平组

我们每天或多或少都会使用社交网络（QQ\空间、微信\朋友圈），通过它，我们可以与他人直接交流、更新自己的状态或者浏览他人的动态等。那么，不管您之前是如何使用社交网络。

现在请您想象一下，在接下来的社交网络使用中，你会经常主动和好友聊天，会主动评论好友发布的动态而不只是浏览它，也会更新自己的动态、上传照片，分享一些东西给好友。总的来说，您在使用社交网络过程中会有很多与好友直接交流的活动。

接下来，也请您回想一下自己最近一次有过以上这些社交网络使用活动，并且尽可能描述更多的细节，例如是主动和朋友聊天还是家人？更新了关于什么的动态？评论了谁的动态？当时的心情如何？或者其他您觉得可以描述的，总之，请您尽量描述多一些。

### 2.主动性社交媒体使用低水平组

我们每天或多或少都会使用社交网络（QQ\空间、微信\朋友圈），通过它，我们可以与他人直接交流、更新自己的状态或者浏览他人的动态等。那么，不管您之前是如何使用社交网络。

现在请您想象一下，在接下来的社交网络使用中，您几乎不怎么会主动和好友聊天，很少主动评论好友发布的动态而只是浏览它，也很少更新自己的动态、上传照片，很少分享一些东西给好友。总的来说，您在使用社交网络过程中很少有与好友直接交流的活动。

#### 操纵检验：

保持想象，对下面的陈述做出评价

①“我会经常在社交网络上进行与好友有直接交流的活动”,“1=完全不同意——7=完全同意”。
